# Laser-Induced Breakdown Spectroscopy: An Efficient Tool for Food Science and Technology (from the Analysis of Martian Rocks to the Analysis of Olive Oil, Honey, Milk, and Other Natural Earth Products)

**DOI:** 10.3390/molecules26164981

**Published:** 2021-08-17

**Authors:** Dimitrios Stefas, Nikolaos Gyftokostas, Eleni Nanou, Panagiotis Kourelias, Stelios Couris

**Affiliations:** 1 Department of Physics, University of Patras, 26504 Patras, Greece; d.stefas@iceht.forth.gr (D.S.); n.guftokostas@iceht.forth.gr (N.G.); e.nanou@iceht.forth.gr (E.N.); p.kourelias@iceht.forth.gr (P.K.); 2 Institute of Chemical Engineering Sciences (ICE-HT), Foundation for Research and Technology-Hellas (FORTH), 26504 Patras, Greece

**Keywords:** Laser-Induced Breakdown Spectroscopy, LIBS, food analysis, machine learning, olive oil, honey, milk

## Abstract

Laser-Induced Breakdown Spectroscopy (LIBS), having reached a level of maturity during the last few years, is generally considered as a very powerful and efficient analytical tool, and it has been proposed for a broad range of applications, extending from space exploration down to terrestrial applications, from cultural heritage to food science and security. Over the last decade, there has been a rapidly growing sub-field concerning the application of LIBS for food analysis, safety, and security, which along with the implementation of machine learning and chemometric algorithms opens new perspectives and possibilities. The present review intends to provide a short overview of the current state-of-the-art research activities concerning the application of LIBS for the analysis of foodstuffs, with the emphasis given to olive oil, honey, and milk.

## 1. Introduction

Laser-Induced Breakdown Spectroscopy (LIBS) has been firstly introduced and proposed for analytical applications almost immediately after the invention of the laser in 1960 [1]. Since then, it has been proposed and today is widely used as an alternative analytical method for numerous applications [2]. The operating principle of LIBS is quite simple and is based on the interaction of a powerful enough laser beam, focused usually on or in a sample, inducing a dielectric breakdown of the material, thus resulting in plasma formation consisting of excited and non-excited atoms and molecules, fragments of molecular species, electrons and ions, and emitting characteristic radiations, whose spectroscopic analysis can in principle provide the elemental composition fingerprint of the material. The required instrumentation consisting basically of a laser source, and a spectrometer/monochromator equipped with the appropriate light detector (nowadays being almost exclusively some CCD or ICCD type detector) is relatively simple and economically affordable, while significant progresses have been achieved to small size and/or portable equipment, facilitating largely the in situ operation [3,4].

The main attributes of LIBS are its capability to provide the simultaneous multi-elemental composition of a sample, of any state of matter (i.e., solid, liquid, or gas, dielectric or conductive), with either little sample preparation or none at all, and due to developments in photonic technology, its instrumentation is of relatively low cost and allows for in situ and on-line applications, as well as applications where the measurement is performed remotely [3]. LIBS-based instruments, either portable or even handheld are now commercialized and are readily available; they are used mainly for the detection of metallic elements in alloys, paints, rocks, soil, etc. [5].

For many years, LIBS was considered mainly for applications related with industrial diagnostic purposes [6]; however, later, it has been applied to environmental, cultural heritage, and space applications [7,8]. Despite the vast field of applications for which LIBS has been proposed, two important issues arise that are considered as drawbacks: the reproducibility and the relatively large detection limits, at least compared with other competing technologies (as e.g., ICP, atomic emission/absorption, etc.). Both these issues are attributed to the relatively lower reproducibility of the laser-induced plasma that take place during the plasma formation [9] and very often to matrix effects [10].

During the past decade, the scientific interest about LIBS-related applications has been importantly revived, mostly due to the implementation of chemometric and machine learning tools for the analysis of the LIBS spectroscopic data [11,12]. In comparison with other spectroscopic techniques, LIBS is superior in terms of collected data, as it can provide enormous datasets with thousands of variables in very short acquisition times. In that sense, the first LIBS benchmark dataset has been published by Képeš et al. [13] which contains LIBS spectra from 138 soil samples belonging to 12 classes. Moreover, based on this benchmark dataset, a comparative classification contest has been performed during the EMSLIBS 2019 conference by Vrábel et al. [14].

Recently, a challenging application of LIBS that received considerable attention is the implementation of LIBS for foodstuff analysis. Specifically, LIBS was employed for the detection of heavy metals in various foodstuffs, for the detection of adulteration and for determining the designation of origin for various types of foodstuffs [15,16]. In particular, during the last decade, more than 100 scientific articles have been published that cover various aspects of LIBS applications in food related analysis. Despite this renewed interest, the related research efforts performed so far concerning the most commonly adulterated foods (i.e., olive oil, honey, and dairy products) are rather limited, to the best of our knowledge.

In the present work, a review is attempted on the recently emerging research and literature regarding the applications of LIBS in the analysis of various types of food, mainly focusing on olive oil, honey, and some dairy products, the main emphasis given to reviewing the chemometric/machine learning methods assisting LIBS analysis. The first section of this review gives a brief overview of the physical phenomena occurring during the implementation of LIBS, as well as a brief overview regarding the related instrumentation. The second section examines some of the most common chemometric and Machine/Deep Learning methodologies that have been applied in order to assist the analysis of LIBS spectroscopic data. Then, the third section presents some recent and innovative research activities of food analysis that employ LIBS. Finally, the last section summarizes some very recent progress concerning LIBS applications for the analysis of olive oil, honey, and dairy products.

## 2. Laser-Induced Breakdown Spectroscopy

**Figure 1:**
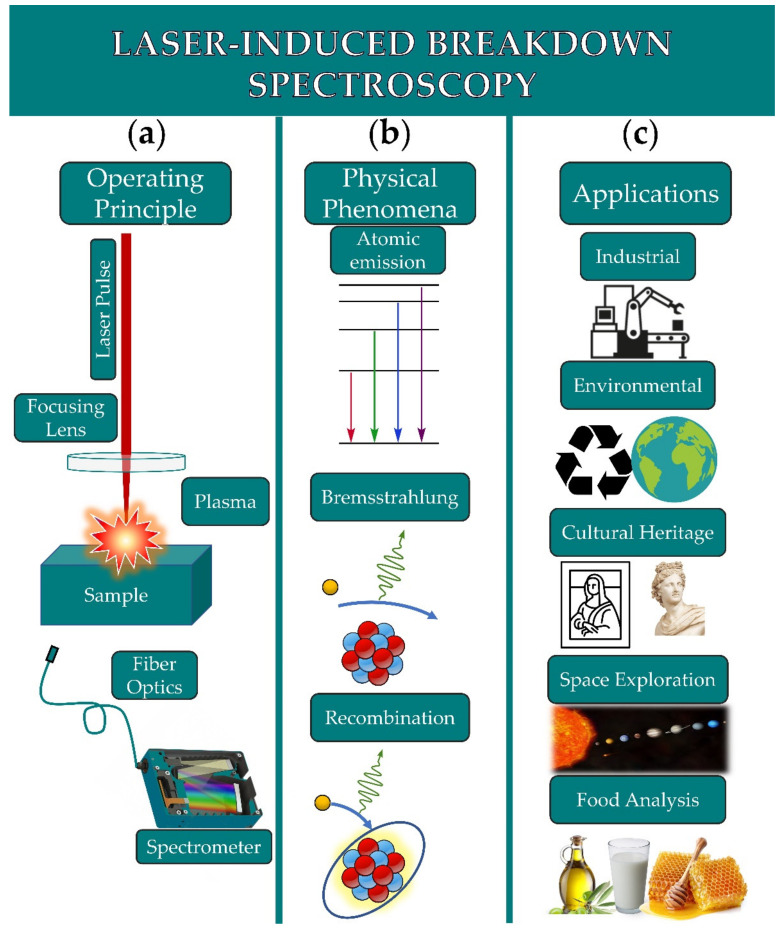
(**a**) Schematic of a LIBS setup. (**b**) Radiative processes occurring in the plasma (atomic emissions, Bremsstrahlung, and recombination). (**c**) Typical applications of LIBS.

LIBS is based on the spectroscopic analysis of the plasma-emitted radiation resulting from the interaction of a strong enough and focused laser beam with a sample. A typical LIBS setup requires a laser, a lens for the focusing of the laser radiation, a lens system or a fiber optic for the collection of radiation emitted from the plasma, and a spectrometer to analyze the collected plasma emission. A schematic of the setup is shown in Figure 1a.

When a strong enough laser beam is focused on the sample’s surface, the material is heated, vaporized, and finally removed; i.e., laser ablation is occurring. The amount of material that is removed depends on the intensity and the wavelength of the laser beam, the laser pulse duration, as well as the material itself (i.e., color, reflectance, surface morphology, etc.). Then, the vaporized ablated material is rapidly expanding above the sample’s surface, forming the so-called high-temperature plasma plume [17].

In general, the laser-induced plasmas contain various species, namely atoms and molecules (excited or not), ions, electrons, and often small diatomic molecules. The latter are either formed following the fragmentation of the sample’s constituents or can be formed through chemical reactions occurring within the plasma volume [18]. The radiation emitted from the plasma usually exhibits several spectral features, arising from radiative transitions of the plasma species, and radiative recombinations and bremsstrahlung, presenting the form of discrete or continuous spectra. Discrete spectra of radiation are due to transitions occurring between bound states of atoms, ions, and molecules. Atomic and ionic transitions exhibit spectral lines, while molecular transitions exhibit band-shaped spectral features. These types of transitions are well studied, and their characteristics (e.g., wavelength, energy levels, etc.) can be found in various databases such as the NIST atomic and molecular spectra database [19,20]. On the other hand, continuous spectra of plasma radiation are due to processes such as Bremsstrahlung and/or to a recombination of electrons with atoms [3,17,18]. A schematic of the atomic and molecular emissions, Bremsstrahlung, and recombination processes are shown in Figure 1b. 

The laser-produced plasmas are in general short-lived, typically lasting up to a few microseconds. Their temporal evolution and analytical description are governed by rather complex dynamics and are out of the scope of this paper. However, for the sake of completeness, a simplistic description of the temporal evolution of the plasma-emitted radiation will be given. So, within few tens of nanoseconds, after the plasma creation, the emitted radiation is basically due to Bremsstrahlung and recombination of electrons with ions, corresponding to a continuous spectrum. A few hundred nanoseconds after the plasma creation, ionic and atomic spectral lines start to emerge and increase in intensity while the continuum decreases rapidly, thus becoming clearly observable, together with the molecular bands.

The analysis and treatment of LIBS data can be demanding, making the extraction of qualitative and/or quantitative information not a trivial task. For instance, LIBS spectra may be complex, containing a large number of features not allowing for the extraction of valuable information about the sample characteristics, whereas spectra from different samples may exhibit the same spectral features, thus impeding the sample discrimination. To overcome such difficulties and by exploiting the ability of LIBS to create large datasets, machine learning and chemometric techniques can be employed. Machine learning algorithms and chemometrics can perform tasks suitable for categorizing/classifying data into desired and distinct classes, calibration measurements, pattern recognition, and outlier detection. These algorithms have been widely applied on LIBS data, for industrial, environmental, cultural heritage, space exploration, and food analysis applications (see, e.g., Figure 1c).

## 3. Chemometrics and Machine/Deep Learning for LIBS

**Figure 2:**
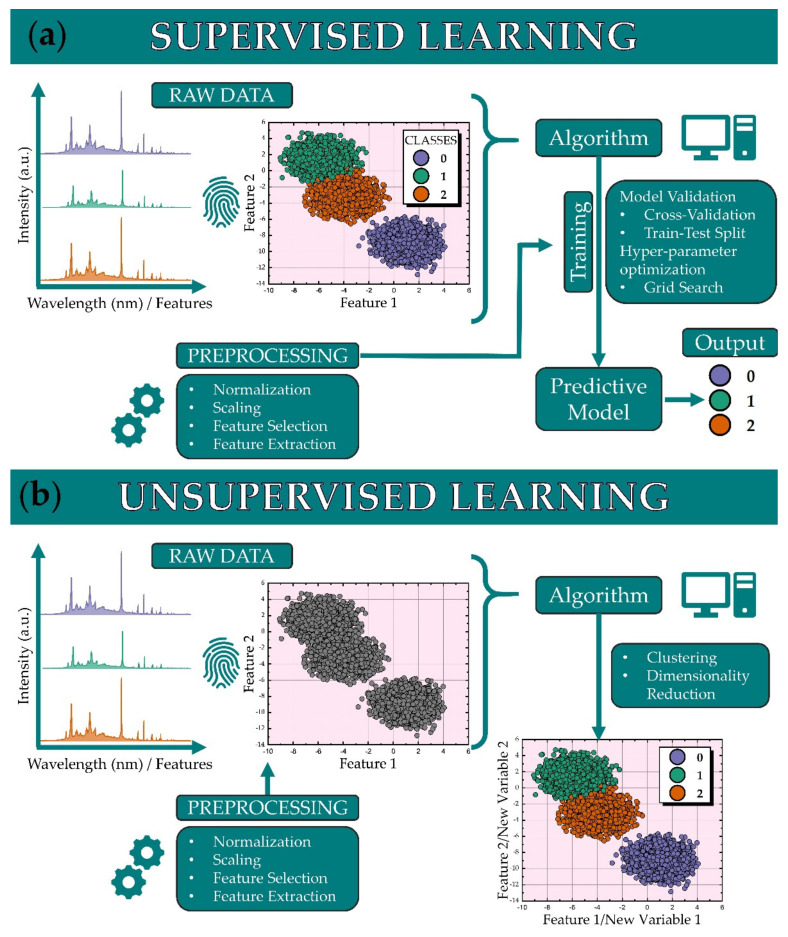
(**a**) Schematic of supervised and (**b**) unsupervised learning algorithmic training.

Chemometric and machine/deep learning algorithms can be categorized into three main classes: supervised, unsupervised, and reinforcement learning. Supervised learning algorithms are employed to associate the data with a specific characteristic/attribute, e.g., a class membership or some continuous value that describes them. Specifically, supervised algorithms are mainly used for classification and regression problems. Unsupervised learning algorithms are used to find patterns within the dataset, for dimensionality reduction, for data clustering, and to detect anomalies [21,22]. Supervised and unsupervised learning approaches adapted to the case of LIBS spectroscopic data are summarized in Figure 2. 

As schematically depicted in Figure 2a, the supervised learning algorithms employ the raw spectroscopic data, i.e., the elemental spectral fingerprints, for which there is a priori knowledge of their class designations. Subsequently, the spectroscopic data are introduced to a machine learning algorithm for training. A validation procedure for the resulting predictive model is usually performed, where the algorithm validates the data introduced to it (by performing cross-validation), and its optimum parameters are determined (by hyper-parameter optimization). It is common practice to pre-process the data, as for example normalizing them, scaling them, as well as selecting certain spectroscopic features (feature selection) or extracting features from them (feature extraction). Common supervised machine learning algorithms that have been used in LIBS studies, are, among others, Support Vector Machines (SVMs) [23,24], Linear Discriminant Analysis (LDA) [25], Partial Least Squares (PLS) [26], Partial Least Squares Discriminant Analysis (PLS-DA) [27], Random Forests (RFs) [28], and k Nearest Neighbors (k-NN) [29]. Moreover, deep learning algorithms have been also used in LIBS studies, with the most popular type being the multi-layer perceptron (MLP) neural networks [12,30].

In contrast to the supervised learning case, in unsupervised learning, there is no a priori knowledge of any class designation [22]. The spectroscopic data are used for training the algorithm that is used to find patterns within the dataset. As a result, the algorithm’s output can be used for various tasks, the most common being clustering and dimensionality reduction (see, e.g., Figure 2b). Common supervised machine learning algorithms that have been used in LIBS studies comprise Principal Component Analysis (PCA) [31] and k-Means Clustering [32], while there have been some works that employ some neural network architectures for unsupervised learning, such as Self-Organizing Maps (SOMs) [33] and Restricted Boltzmann Machines (RBMs) [34]. Less commonly, graph theory-based algorithms have been also used for the treatment of LIBS spectra in an unsupervised manner, with impressive results [35].

**Figure 3:**
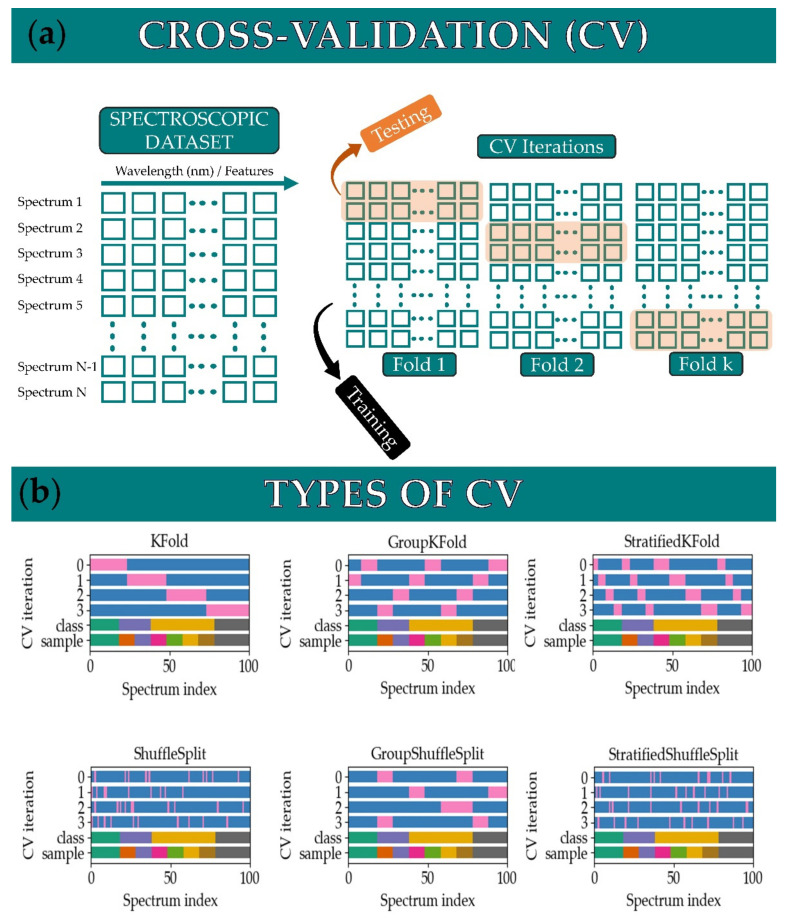
(**a**) Schematic of cross-validation. (**b**) Types of cross-validation (Adapted from ref. [37]).

An important issue, often omitted however, regarding the algorithmic training for supervised algorithms is the validation of the predictive models. The most common method is cross-validation (also known as internal validation), which is denoted as CV in the rest of this work. CV is used to assess how a predictive model can generalize to an independent dataset (i.e., to assess if there is any bias in prediction that is due to either the training procedure or the data) and is schematically explained in Figure 3. During the training of the spectroscopic data, CV is used to split it into training and testing sets. In the related literature, its implementation appears in many variants [36]. The simplest form of CV is to simply split the data into two subsets and use one for training and the other one for testing. The most commonly used type of CV is the k-fold CV, where the entire dataset is split into k subsets and the algorithm is iteratively (k times) trained with the k-1 subsets, while the remaining dataset is used for testing purposes. The procedure is schematically depicted in Figure 3a. A value between 5 and 10 has been shown that is the most suitable for k [36]. This can be seen in Figure 3b, where a five-fold CV is used to train a dataset consisting of 100 spectra (bottom axis stands for the spectrum index), from eight samples that belong to three distinct classes. For each CV iteration, the subset used for training is blue colored, while the subset used for testing appears as pink colored.

Some other variants of CV, which are not used so often in treating LIBS spectroscopic data, are shown in the rest of Figure 3. These are group k-fold (in which the same group is not appearing in two different folds), stratified k-fold (where the folds are selected so that the mean response value is approximately equal in all the folds), shuffle-split (random selection of the train and test sets and does not guarantee that all folds will be different, especially for relatively small datasets), group shuffle-split (provides randomized train/test indices to split data according to a third-party provided group), and stratified shuffle-split (is a merge of stratified k-fold and shuffle-split, which returns stratified randomized folds. The folds are made by preserving the percentage of samples for each class.). These CV variants are readily implemented in most of the available chemometrics and machine learning libraries and packages [37].

A rather important and common problem may occur when performing CV that concerns the data itself and the implementation of CV, as well, i.e., data leakage. Data leakage means that information is revealed to the predictive model in such way that it attributes it with an unrealistic advantage to make better predictions. This leakage occurs, mainly, at the pre-processing steps where normalization/standardization and/or dimensionality reduction are applied to the data. The aforementioned steps must be performed after the data splitting to the training and test set; otherwise, data leakage will occur, and non-trustworthy results will be obtained.

Accordingly, for the proper evaluation of a constructed algorithmic model, internal validation (i.e., cross-validation) must be accompanied with an external validation as the final step. In order to perform an external validation, a set of samples must be excluded totally from the training procedure and used only for prediction purposes after the algorithmic training. This is the only way to make sure that the constructed predictive model is well-trained, effective, and robust.

## 4. LIBS Applications in Food Analysis

Over the last few years, Laser-Induced Breakdown Spectroscopy (LIBS) has demonstrated its potential as a useful spectroscopic tool for food analysis and diagnostics [15,16]. Along with the plethora of the food products found in the market and the issues regarding food safety, a significant number of studies have appeared using LIBS. Some of these studies that have been conducted very recently, after 2019, are briefly described below.

A rather interesting topic of food science is the food products obtained from genetically modified organisms and crops. These types of foodstuff (the most common being corn, soy, zucchini, milk, etc.) need to follow specific regulations and guidelines before being allowed on the market. Considering the above, in an interesting work from Liu et al. [38], LIBS was combined with machine learning techniques, including Principal Component Analysis (PCA) and Partial Least Squares Discriminant Analysis (PLS-DA), for discriminating genetically modified maize. As a result, the discrimination of 120 transgenic and 120 non-transgenic maize samples with 100% accuracy has been succeeded, and spectral features of high importance have been identified and were reported. 

Another topic of interest is the detection of heavy metals, pesticides, fungicides, and toxic substances in foodstuff. In a recent study by Wang et al. [39], cadmium (Cd) content on rice roots was determined by LIBS assisted by different machine learning algorithms (i.e., Partial Least Squares Regression (PLSR), Least Square Support Vector Machines (LS-SVM), and Extreme Learning Machine (ELM)). The investigation was focused on the spectral lines of atomic and ionic cadmium (Cd (I) at 228.80 nm, Cd (II) at 214.44 and 226.50 nm). In comparison with conventional chemical procedures, such as ICP-OES, LIBS performed rapid analysis, much faster that ICP-OES, thus being an attractive method for real-time analysis of crops. In the same direction, Wu et al. [40] used LIBS aided by Hyperspectral Imaging (HSI) in order to perform fast detection of thiophanate-methyl on the surface of some mulberry fruits, employing PCA and PLSR analysis. Thiophanate-methyl is a fungicide, i.e., a biological chemical compound, often used for exterminating several parasites in a large variety of crops. In another study by Gamela et al. [41], ICP-OES and LIBS have been used for the quantitative determination of the concentration of elements, such as Ca, K, Mg, Na, and P, in edible seeds. In addition to LIBS and ICP-OES, hyperspectral imaging was used for mapping the presence of the aforementioned elements on the seeds’ surface or inside them. Larios et al. [42] discriminated low and high-vigor soybean seed lots, using LIBS along with machine learning techniques such as Principal Components Analysis (PCA), Support Vector Machines (SVMs), Linear Discriminant Analysis (LDA), Quadratic Discriminant Analysis (QDA), and k-Nearest Neighbors (k-NN). Furthermore, LIBS has been used for discrimination of both the cultivar [43,44] and geographical origin [45] of different kinds of foodstuffs. In that view, Perez-Rodriguez et al. [43], after selecting the emission lines of carbon (C), calcium (Ca), iron (Fe), magnesium (Mg), and sodium (Na) and using the Extreme Gradient Boosting (XGBoost) algorithm, developed a k-NN model able to predict the cultivar of brown rice with accuracy up to 86%. Similarly, Megalhães et al. [44] used LIBS combined with some machine learning algorithms (i.e., PCA, PLSR, etc.) and achieved the successful discrimination of some sweet oranges, with similar DNA, with high accuracy. Finally, Zhang et al. [45] have succeeded in classifying some Ginkgo biloba leaves based on their geographical origins utilizing the LIBS technique aided by some machine learning algorithms (PCA, LDA, and SVM).

From this short literature review of the very recent LIBS-related applications concerning foodstuffs, it becomes clear that LIBS applications are rapidly expanding in food science and technology, and that LIBS is gradually established as an attractive diagnostic tool for foodstuffs and related security issues. In the next section, we will focus on related research that concerns some foodstuffs that are very common and very largely consumed and thus, their quality control and safety are of great importance and of high socioeconomic implications. Such foods are the edible oil and its various types, the honey and the milk.

### 4.1. Olive Oil

Olive oil is a highly reputable foodstuff mainly produced and consumed within Mediterranean countries (e.g., Spain, Italy, Greece), and its use is widely spread to several countries around the world. In comparison with other vegetable oils, olive oil stands out for its unique taste and color, delicate aroma, and its potential health and therapeutic benefits [46,47,48]. High-quality olive oils are branded as extra-virgin (EVOO) and virgin (VOO), while monovarietal olive oils have unique characteristics resulting from the olive cultivar, geographical origin, and climatic conditions. The knowledge of cultivar and geographical origin can be of high commercial interest, especially for premium olive oils that can bear marks such as the protected designation of origin (PDO), protected geographical indication (PGI), and traditional specialty guaranteed (TSG). However, olive oil is a food prone to food fraud such as mislabeling and adulteration due to its increasing demand and higher market values, especially for EVOOs and VOOs.

In that view, a plethora of analytical techniques such as HPLC, FT-IR, and NMR [49,50,51,52] have been applied in recent years not only to study the adulteration of olive oil but also for the verification of its geographical origin and variety, its content of fatty acids (palmitic, oleic, linoleic, and linolenic) and/or several other compounds, such as polyphenols, tocopherols, sterols, etc. However, although these techniques exhibit high sensitivity, they are costly in terms of equipment and usually demand highly qualified personnel for their operation, while they require time-consuming procedures for sample preparation. Therefore, a rapid, lower cost, reliable, and accurate enough method can be of great interest and importance for olive oil quality control and other analysis needs. The LIBS technique fulfills the above-mentioned requirements and recently has been proposed to be applied for olive oil discrimination/classification issues, providing so far very promising results. In this review, we focus on the application of the LIBS technique for the discrimination/classification of olive oil in terms of geographical origin, type of cultivar, and adulteration with other vegetable much cheaper oils. 

**Figure 4:**
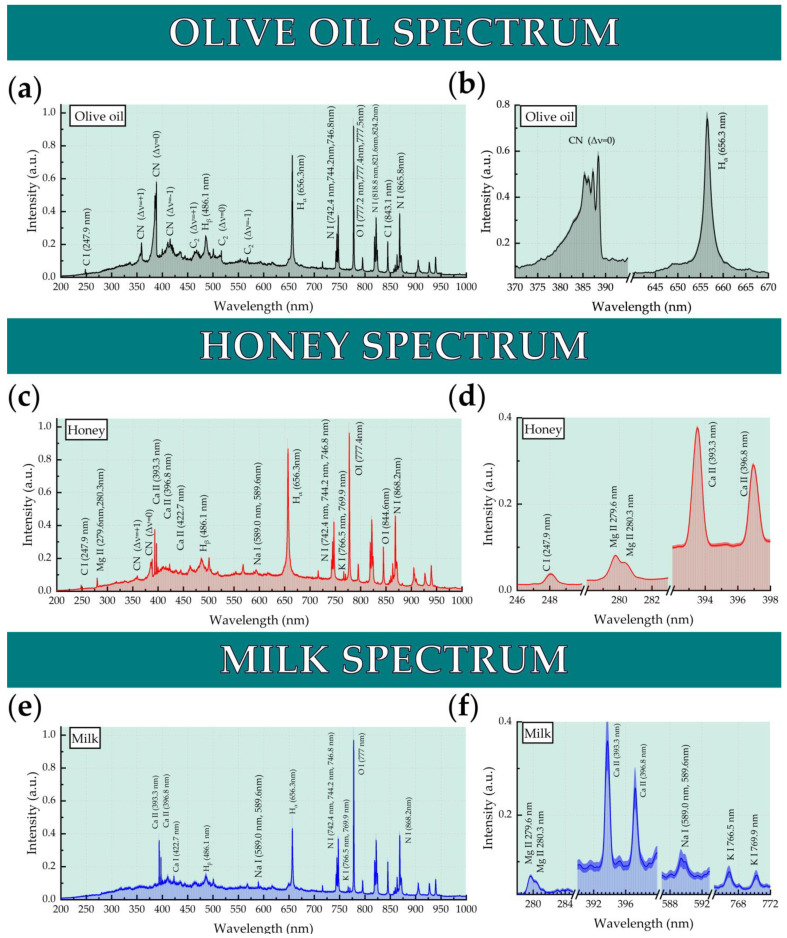
(**a**)Typical LIBS spectrum of olive oil. (**b**) Enlarged view of the olive oil spectrum indicating the CN band and the H_α_ line. (**c**) Typical LIBS spectrum of honey. (**d**) Enlarged view of the honey LIBS spectrum indicating the atomic line of C and the ionic lines of Mg and Ca. (**e**) Typical LIBS spectrum of milk. (**f**) Enlarged view of the milk LIBS spectrum indicating the ionic lines of Mg and Ca and the atomic lines of Na and K.

In Figure 4a, a representative LIBS spectrum of an olive oil is shown. As shown, it contains the typical spectral features of elements found in organic matter, i.e., carbon (C), nitrogen (N), hydrogen (H), and oxygen (O), as well as the molecular bands of CN and C_2_. On the Figure 4b, an enlarged view of the CN band’s Δν = 0 and H_α_ Balmer line are shown. 

To the best of our knowledge, the work by Caceres et al. [53] is the first one using LIBS for olive oil analysis. In this work, different types of edible oils (olive, sunflower, hazelnut, and corn oil) from four different countries were studied, and a total of 118 oil samples were studied by means of a neural network algorithm for classification purposes, achieving accuracies as high as 95%.

In another work, Kongbonga et al. [54] studied the classification of some vegetable oils (extra virgin olive oil, refined sunflower oil, refined and crude palm oil, and refined corn oil) based on their saturated fatty acids content. For this purpose, they studied the spectral features of edible oils and correlated the LIBS signal with the saturated fatty acids content of the samples. Interestingly, a relationship between the oils’ saturated fatty acid content and the C_2_ molecular emissions was reported to hold. One-way analysis of variance (one-way ANOVA) was performed using the ratio of the emission intensities of the C_2_ band and the C (I) 247.9 nm spectral line and a Tukey’s honest significance test was performed to determine which vegetable oils, from the studied ones, were significantly different from the others.

Gazeli et al. [55], suggested, as a proof of concept, the capability of LIBS to classify olive oil samples of different acidities and designation of origin. The major spectral features of olive oil LIBS spectra were thoroughly discussed, and various machine learning algorithms were used to classify these spectra, i.e., Linear Discriminant Analysis, Support Vector Machines, and Random Forests. It was shown that Principal Component Analysis can indicate the variance of each spectral feature, while it can be used as a feature extraction method as well by using the obtained principal components as inputs to the machine learning algorithms. Using this methodology, high classification accuracies were obtained, with LDA being the most successful algorithm, attaining a (99.2 ± 1.5)% correct classification. The same methodology was applied in another work by Bellou et al. [56], more focusing on the effect of the experimental conditions on the clustering and classification of the olive oil LIBS spectroscopic data. In that view, three different experimental configurations for handling of olive oil samples were employed and tested, where the laser-induced plasma was created in an olive oil spray, on a thin laminar flow of olive oil and on the free surface of few grams of (liquid) olive oil. In addition, the effect of the experimental parameters (i.e., laser energy, gating parameters, etc.) on the collected LIBS spectra and their subsequent classification by PCA and LDA were thoroughly investigated and assessed.

In another work by Gyftokostas et al. (2020) [57], LIBS assisted by several different machine learning algorithms, namely, Linear Discriminant Analysis (LDA), k-Nearest Neighbors (k-NN), and Support Vector Classifiers (SVC)) was applied for the classification of some olive oils from different regions of Crete island, based on their designation of origin. In addition, in this work, the Principal Component Analysis (PCA) was assessed as a feature extraction method, which can assist the classification procedure as it retains information regarding spectral features with high variance. Comparative assessment of the various algorithms was performed for both PCA-processed and raw data. The optimal model was found to be LDA combined with pre-processing using PCA to focus on the variables with the highest discrimination capability, minimizing the computational time required for the model. Despite the relatively small geographical footprint, a discrimination accuracy of (94.0 ± 1.1)% was achieved.

In another work, Gyftokostas et al. (2021) [58] performed classification of olive oil LIBS spectra based on their designation of origin and employed some machine learning algorithms to determine the importance of their spectral features. More specifically, 139 extra virgin and virgin olive oil samples were studied, originating from three different regions of Greece, i.e., Peloponnese, Crete, and Lesvos. The algorithms that have been employed were Linear Discriminant Analysis (LDA), Extremely Randomized Trees Classifier (ERTC), Random Forest Classifier (RFC), and eXtreme Gradient Boosting Classifier (XGBoost). ERTC, RFC, and XGBoost being ensemble methods were used both as classifiers and as feature selection algorithms that reveal the importance of each spectral feature. Spectral features of carbon (C), oxygen (O), and nitrogen (N) spectral lines, as well as CN and C_2_ molecular bands were found to contribute the most to the classification. An important parameter that has been carefully examined in this work was the effect of the number of important features on the classification accuracy. Although all employed algorithms attained high classification accuracies, the most successful model was found to be the XGBoost one, since it succeeded in both reducing the initial dataset by thousands of times, retaining only two spectral features, while maintaining a very high accuracy up to 99%. 

In another very recent study by Gyftokostas et al. (2021) [59], the classification of olive oil samples based on their geographical origin was studied employing two spectroscopic techniques, namely LIBS and absorption spectroscopy. The former technique provided the emission spectrum revealing the elemental composition of the olive oil samples, while the latter one provides spectroscopic information related to the chlorophylls and carotenoids content of the olive oil sample, corresponding to the absorption bands appearing within the 350–750 nm spectral region. In this comparative study 143 Greek olive oil samples from three different regions were studied. The study included mixtures of samples with different origins as well. Both emission (i.e., LIBS spectra) and absorption spectra were initially pre-treated by Principal Component Analysis (PCA) and then were introduced in the algorithms of Linear Discriminant Analysis (LDA) and Support Vector Classifiers (SVC). For each spectroscopic method, the number of PCs that provide the most efficient classification accuracy was assessed. The accuracies obtained were as high as 100% with the algorithmic training being evaluated and tested by means of classification reports, confusion matrices, and by external validation procedure as well. 

As the instrumentation of both LIBS and absorption spectroscopy has reached a high level of maturity, both methods having low operational costs, with the latter being much more widely spread and routinely used in labs, the combination of the datasets each one provides was attempted very recently. So, in the work by Stefas et al. [60], as a natural continuation of the work of ref. [59], data fusion of LIBS and absorption spectroscopic data was performed for the discrimination of olive oils based on the olive cultivar origin. In that view, LIBS and absorption spectra were collected from two different olive oil cultivars that are quite wide spreading and popular in Greece, namely the Koroneiki and Kolovi cultivars. The two types of spectra were fused appropriately and used to develop classification schemes employing Linear Discriminant Analysis and Gradient Boosting, the latter allowing the computation of feature importance. The resulting models contained spectroscopic information regarding the emission and absorption profiles of each sample (i.e., elemental composition and carotenoid/chlorophyl content). The obtained accuracy was found exceeding 90%, suggesting that data fusion can significantly improve the correct classification of olive oils being a reliable innovative approach.

### 4.2. Honey

Honey is the sweet viscous substance made by bees that collect nectar from the sweet secretions of trees and plants. Its major constituents are carbohydrates and water, with the main types of carbohydrates being sugars, i.e., fructose, glucose, and sucrose. It also contains several other substances at low concentrations, such as proteins, vitamins, minerals, salts of organic acids, and naturally occurring trace elements. Since its composition is related with various health benefits, honey usually attains high prices in the market, and because it is easy to adulterate with other types of syrup, or lower quality honey, it is inevitably one of the most commonly adulterated foods [61]. LIBS has been recently proposed as an alternative technique to assess honey quality, such as for detecting adulteration and determining its botanic and geographic origin.

In Figure 4c, a representative LIBS spectrum of honey is shown. As can be seen, it exhibits the usual spectral signatures of elements commonly found in the LIBS spectra of organic matter, i.e., carbon (C), nitrogen (N), hydrogen (H), and oxygen (O), and the molecular bands of CN as well. Moreover, honey LIBS spectra exhibit the spectral lines of several inorganic species, such as magnesium (Mg), calcium (Ca), sodium (Na), and potassium (K). On Figure 4d, an enlarged view of the spectral lines of these inorganic species are shown. 

Recently, Stefas et al. [62] studied the characteristics of the plasma created on some honey samples and the temporal evolution of the spectral lines of the inorganic ingredients of honey, among other things, in view of optimizing the experimental conditions to achieve high classification accuracy of honey samples based on their floral origin. For the classification of the honey LIBS data, several machine learning algorithms were utilized such as Principal Component Analysis (PCA), Linear Discriminant Analysis (LDA), Support Vector Machines (SVMs), and Random Forest Classifiers (RFCs). The implementation of PCA on the acquired LIBS data showed a clear discrimination between the ten different types of honey studied, while the PCA loadings showed the features with the largest variance, namely the emissions of oxygen (O), nitrogen (N), potassium (K), hydrogen (H), magnesium (Mg), calcium (Ca), sodium (Na), as well as the CN molecular band. The implementation of Random Forests indicated the feature importance of K, Mg, Ca, and Na spectral lines. Moreover, the constructed models using both the LDA and SVC algorithms, carried out the classification of the samples of different botanical origin, with accuracy of 99.8%, while the RFC model had an accuracy of 97.8%. In a similar work, Se et al. [63] created a Partial Least Squares Regression model predicting the concentration of calcium (Ca), magnesium (Mg), and sodium (Na) by using LIBS spectra from 30 stingless bee honey samples. The constructed PLSR model was subsequently evaluated by inductively coupled plasma-optical emission spectrometry (ICP-OES) measurements in order to determine the concentrations of Na, Ca, and Mg. The effectiveness of the predictive model was tested by three kinds of tests, including coefficient of determination R-square (R2), standard error of calibration (SEC), and standard error of cross validation (SECV). In another work, Nespeca et al. [64] performed the classification and regression of the LIBS spectra of some adulterated honey samples by means of Partial Least Squares Discriminant Analysis (PLS-DA) and Partial Least Squares Regression (PLS), respectively. Specifically, after performing LIBS assisted by spark discharge (SD-LIBS) on 236 unadulterated and adulterated honey samples, PLS-DA was used to identify the type of the adulterant and PLS was used to quantify the percentage (%) of adulteration. In more detail, the honey samples used were from three botanical origins, i.e., Eucalyptus, Citrus sinensis, and multifloral, and the adulterants were high-fructose corn syrup (HFCS) and sugar cane syrup (molasses). To improve the performance of the PLS and PLS-DA models and reduce their complexity, the SD-LIBS data were pre-processed with various methods and were evaluated by means of cross-validation, external validation, and other methods as well. After that, variable selection was performed and assessed with different methods including the interval partial least squares (iPLS) and some genetic algorithms (GA), concluding that the most important emissions are those arising from the inorganic species of honey, such as those of Ca and Fe. In the same spirit, Lastra-Mejías et al. [65] used LIBS assisted by k-NN algorithm in order to classify some honey samples adulterated with rice syrup. As a feature selection method, a relief-based algorithm was chosen which results to a set of chaotic parameters extracted from the LIBS spectra. In another work, Peng et al. [66] created a PLS regression model using the LIBS spectra of adulterated acacia and rape honey samples by two kinds of high-fructose corn syrups, focusing on the aforementioned, spectral features of the inorganic ingredients of honey (i.e., Mg, Ca, Na, and K). Zhao et al. focused on the discrimination of honeys originating from different geographical regions [67] and achieved the classification of 120 acacia honey samples from three different geographical regions, utilizing SVM and LDA algorithms after pre-treatment of the data using PCA. Moreover, one-way ANOVA was performed using the intensities of the various spectral features to investigate whether they can be used for the geographical discrimination of honeys. In another very recent work by Stefas et al. [68], the utility of LIBS for the detection of honey adulteration with some glucose syrup via LDA and Extremely Randomized Trees algorithms was demonstrated. In there, it was shown that instead of the entire LIBS spectrum, only the spectral lines of the inorganic ingredients of honey (i.e., calcium, sodium, and potassium) can be employed for the successful detection of adulteration, with classification accuracies exceeding 90% of correct classification.

### 4.3. Dairy Products 

Undeniably, milk is among the most important foods. Not only because of its nutritional value, but because it is the primary food that mammals need from the time they are born. Most importantly, in the case of babies, breast milk is essential for the healthy growth and the formation of antibodies. Regarding the nutritional values, milk and milk products are rich in protein, fat, and carbohydrate and are a very important source of nutrients, including calcium, phosphorous, potassium, magnesium, etc. [69].

In Figure 4e, a representative LIBS spectrum of milk is shown. As shown, it contains several spectral features, corresponding to nitrogen (N), hydrogen (H), and oxygen (O), while several other spectral lines attributed to its inorganic ingredients are also prominent, as e.g., those of magnesium (Mg), calcium (Ca), sodium (Na), and potassium (K). An enlarged view of these spectral lines is shown in Figure 4f. Therefore, it is obvious that these spectral lines will be of interest for milk analysis via LIBS.

To the best of our knowledge, the work of Abdel-Salam et al. [70] is most probably the first study about milks and dairy products employing LIBS. In this work, a comparison of maternal milk and commercial infant formulas is presented, studying milks from mothers and commercial infant formulas, which are suitable for newborns (<3 months). From the acquired LIBS spectra, correlations between maternal milk and infant formulas were found. The study showed that trace elements, such as magnesium (Mg), calcium (Ca), sodium (Na), and iron (Fe), appeared with stronger intensities in infant formulas than in maternal milk. The same pattern was reported for the molecular bands of CN and C_2_. Next, in a continuation of the previous work, Abdel-Salam et al. [71] studied the different farm animals’ milk, as e.g., buffalo, camel, goat, and sheep milks; their analysis focusing on the spectral lines of magnesium (Mg), calcium (Ca), sodium (Na), iron (Fe), strontium (Sr), and barium (Ba). According to this report, the spectral lines of Mg and Fe were found to be stronger in goat milk, while Ca and Sr spectral lines were found to be stronger in camel milk. The same trend was also reported for the Ba and Na lines in buffalo milk. In addition, the CN band, correlated to the protein content (e.g., casein), was found to be stronger in camel milk, suggesting a higher concentration of proteins in this type of milk. Along the same lines, Abdel-Salam et al. [72,73] used LIBS to discriminate milk samples from healthy cows and cows suffering from mastitis, the latter being an inflammatory infection, which has serious implications on milk, breaking down its proteins. So, in ref. [72], two different spectroscopic techniques were used, namely LIBS and LIF (Laser-Induced Fluorescence), and several milk samples from healthy cows and from cows with observable signs of mastitis were studied. Again, significant variation of the Ca and Na spectral lines intensities were reported between healthy cows and cows suffering from mastitis, the Ca emissions being stronger in healthy cows, while the Na emissions were stronger in cows with mastitis. In a similar study [73], the CN and C_2_ emissions were used for the discrimination of milk from healthy and mastitis’ suffering cows. In another work from the same group [74], LIBS was employed assisted by Principal Component Analysis (PCA) for the discrimination between colostrum and mature sheep milk. The analysis of LIBS spectra suggested a reduction of the intensities of the Ca, CN, and C_2_ emissions, correlated to the degradation of proteins, vitamins, lactoferrin, minerals, etc. which are characteristic ingredients in sheep colostrum milk.

In a different direction, Bilge et al. [75] investigated the use of LIBS for the detection of milk adulteration. Specifically, they employed LIBS implementing some PCA algorithms to discriminate between non-adulterated milk powder and milk powder adulterated with sweet and acid whey powders. Moreover, using some Partial Least Squares (PLS) models, they have constructed calibration curves and reported limits of detection (LOD) of 1.55 and 0.55% for adulteration with sweet whey powder and acid whey powder respectively.

In another work, Cama-Moncunill et al. [76] studied the quantification of trace metals, such as copper (Cu) and iron (Fe), in some infant formula premixes with lactose and various minerals of Cu and Fe employing LIBS and some linear, multilinear, and partial least squares regression analysis methodologies. The best results were obtained by partial least squares regression and were validated by atomic absorption measurements. 

Similarly, Chen et al. [77] employed LIBS assisted by some chemometric algorithms for the quantification of potassium (K) in infant formulas, validating the results by atomic absorption. Based on these measurements, a predictive model based on partial least squares has been constructed. The spectra were pre-processed by a novel strategy that included normalization of the data, a wavelet transform, and feature selection based on the random frog algorithm.

In the same spirit, Lei et al. [78], used Calibration-Free LIBS (CF-LIBS) for the determination of the concentration of some mineral elements, such as calcium (Ca), magnesium (Mg), and potassium (K), in powder milks. The determined concentrations were validated by inductively coupled plasma atomic emission spectroscopy (ICP-AES). Employing the same methodology, Rehan et al. [79] studied the determination of the content of several elements in milk powder samples by LIBS, including elements such as cadmium (Cd), chromium (Cr), copper (Cu), nickel (Ni), manganese (Mn), iron (Fe), aluminum (Al), sodium (Na), phosphorous (P), sulfur (S), and zinc (Zn).

In a different direction, aiming to fraud detection, Moncayo et al. [80] studied two common practices of milk adulteration. Specifically, the mixing of milk of different animal origin and the adulteration of milk powder with melamine were examined. In the first case, cow, goat, and sheep milk were used to create binary and ternary mixtures in various concentrations. Then, the obtained LIBS spectra were classified into three classes, i.e., pure milks, binary mixtures, and ternary mixtures, by employing principal component analysis and multilayer perceptron neural networks, with a classification accuracy of 100%. In the second case, mixtures of milk powder with melamine were created, and a calibration curve was constructed correlating the CN band emission with the adulteration content. Specifically, it was reported that the CN band’s intensity increased with the addition of melamine in the milk sample. In addition, by analyzing the spectroscopic data by some neural networks, the prediction of the adulteration content was succeeded with correlation coefficients as high as 0.999. 

Finally, for the detection of fraud, Sezer et al. [81] used LIBS coupled with some chemometrics algorithms for determining the ratio of milk mixtures from different animal origins as well as to discriminate between milks of different animal origins. Specifically, spectra of ovine–bovine and caprine–bovine binary mixtures were analyzed successfully by partial least squares and the discrimination of ovine, caprine, and bovine samples was performed successfully by means of principal component analysis.

## 5. Conclusions

LIBS, being a simple, versatile, and powerful technique, has gained significant popularity during the last decades, and it has been proposed for a plethora of applications in different scientific fields. It is only during the last few years, that LIBS applications for food analysis-related applications have emerged. However, this advancement would have remained limited unless modern machine learning and chemometrics methods are implemented, as the complexity of organic matter and the similarities of the respective LIBS spectra do not allow for safe conclusions. So, LIBS assisted by machine learning and chemometrics started to become an accepted method for food control and quality assurance. In the present review, the current state-of-the-art of food-related analysis via LIBS aided by machine learning algorithms is briefly presented. Finally, a special emphasis is given for the applications of LIBS assisted by machine learning for the analysis of LIBS spectroscopic data, for olive oil, honey, and dairy products, which are essential foodstuffs for human nutrition and health, and thus, they have important societal and economic implications and impact.

## Data Availability

Not applicable.
